# Epidermoid Cyst of the Uterine Cervix, an Unusual Location: Literature Review and Case Report

**DOI:** 10.3390/healthcare11020257

**Published:** 2023-01-13

**Authors:** Camelia Liana Buhas, Andrei Pascalau, Claudia Teodora Judea-Pusta, Ovidiu Laurean Pop, Adrian Sorin Judea, Bianca-Maria Negrutiu, Lavinia Marcut, Bogdan Adrian Buhas, Daniela Gheorghita, Alin Danut Bodog

**Affiliations:** 1 Department of Morphological Sciences, Faculty of Medicine and Pharmacy, University of Oradea, 410073 Oradea, Romania; 2 Department of Dental Medicine, Faculty of Medicine and Pharmacy, University of Oradea, 410073 Oradea, Romania; 3 Department of Surgical Sciences, Faculty of Medicine and Pharmacy, University of Oradea, 410073 Oradea, Romania; 4 Oradea County Emergency Clinical Hospital, 410169 Oradea, Romania; 5 Faculty of Materials Science and Engineering, University Politehnica of Bucharest, 060042 Bucharest, Romania

**Keywords:** epidermoid cyst, uterine cervix, immunohistochemistry, HPV

## Abstract

Epidermoid cysts are most often benign cystic lesions, with uterine cervical localisation being very unusual. We present the case of a 52-year-old female patient diagnosed with an epidermoid cyst at the level of the uterine cervix. A bioptic and haemostatic uterine curettage was performed, followed by total hysterectomy with bilateral adnexectomy. The histopathologic analysis and immunohistochemical essay of the resection specimens confirmed the cervical epidermoid cyst. The presence of high-risk HPV (human papillomavirus) was only seen in the cervical mucosa. The exact etiopathogenesis is unknown, but postpartum cell implantation of reminiscent embryonic tissue can be involved in the development of these lesions.

## 1. Introduction

Epidermoid cysts are most often benign cystic lesions, commonly seen on the face, scalp and trunk, and rarely can occur on the glabrous skin of the palm and sole and genital organs [1,2,3,4]. To date, we feel that there is still some confusion in the specialist literature because many still use inaccurate terms to identify cysts, such as epidermoid cysts that are also called epidermal, infundibular or keratinous cysts. Looking back in time, we see that keratinous cysts have been known for many years as sebaceous cysts, a misnomer born of a mistaken gross interpretation of the cyst content and perpetuated by uncritical repetition [1,3].

Probably the simplest and most accurate way to describe such lesions was proposed by Ackerman [1], who speaks of two types of keratinous cyst, with the occasional possible occurrence of hybrid forms. The most common (90%), known as the epidermal or epidermoid type, is lined by cornified epithelium, has a distinct granular layer, and contains lamellated keratin without calcification [1,5]. 

Although some of these cysts (particularly those located in the fingers) result from traumatic inclusion of the epidermis—hence the term epidermal inclusion cyst—the majority probably arise from the cystic dilation of the infundibular portion of the hair follicles [6]. A few of these cysts exhibit seborrheic keratosis-like changes in their wall [1]. According to a study performed by Choi et al. [6], using 3D reconstruction analysis, the plantar epidermal cyst may be connected to eccrine dermal ducts, supporting the hypothesis that certain plantar epidermal cysts develop from the epidermoid metaplasia of eccrine ducts.

HPV (human papillomavirus) (usually type 57 or 60) has been found in cases of keratinous cysts of palmoplantar and other locations, suggesting that it may play an etiological role [1,6,7,8,9].

The other type of keratinous cyst is the pillar or trichilemmal type. It occurs preferentially on the scalp and is microscopically characterised by a trichilemmal type keratinization, which is a sudden keratinization process without the formation of a granular layer and an uneven interphase between the keratinised and non-keratinised cells. The keratin inside the cyst is not lamellated, some of the nuclei are retained and focal calcification is frequent. Ultrastructural and immunohistochemical studies also support a trichilemmal derivation for this lesion [1]. Epidermoid cysts in the kidney with nephrolithiasis have rarely been reported, and in most cases, its pathogenesis has not been well understood, although the chronic irritation induced by renal stones may be a risk factor [10]. A case report published in 2015 by Pehlivan et al. [11] presented these types of lesions in the labia minora. Some others case reports show epidermal cysts in the clitoris or labia majora, being very uncommon in adolescent girls [3,4,11,12,13,14,15].

A particularly rare localisation for an epidermoid cyst is represented by the uterine cervix. A survey of the specialized literature brings to our attention only one other such case, published in 1925 by Bacon [16]. Willson and Cimon describe a Cervix—Squamous Cyst in a female F344/N rat from a chronic study [17,18,19].

## 2. Case Report

A female patient, aged 52, comes to the obstetrics–gynaecology clinic for haemorrhagic metropathy and fatigue. A bioptic and haemostatic uterine curettage was performed, and the histopathological analysis shows an endometrial atypical hyperplasia/endometrioid intraepithelial neoplasia (EAH/EIN) with focal atypia. After the prescribed treatment, there were no notable results; as a consequence, it was decided to radicalise the intervention and perform a complete hysterectomy with bilateral adnexectomy. 

The macroscopic examination of the surgical piece was performed revealing a polypoid grey mass with brown areas in the endometrial cavity, overall thickened myometrium, two ovarian cysts less than 3 cm in diameter with vitrine serous content and smooth inner surface, the same at the fallopian tube level, some brown areas at the surface of the cervix less than 2 mm each in diameter and a conspicuous cyst filled with a fragile grey substance of 1.8 cm in diameter located at relative deeper layer of the exo-cervix. 

The primary sections were stained with haematoxylin–eosin (HE). An immunohistochemical analysis was performed on sections prepared from formalin-fixed paraffin embedded tissue using an automated immune-stainer (BechMark GX, Ventana Medical Systems Inc., Tucson, AZ, USA). Immunohistochemical assays were performed on a Ventana Benchmark GX automated staining instrument according to the manufacturer’s instructions [20,21,22]. For the immunohistochemical techniques to identify the squamous epithelium of the cyst wall, we used CK 5/6(D5/16B4 clone) and p16 (E6H4 clone), provided by Ventana Medical Systems, Inc., and they showed a colour reaction in brown. For both of the markers, a positive control sample was used using tonsil tissue [20]. The negative control was carried out in the same way, but with primary antibody omission. The Leika 3000 DM with a high-definition (HD) photo camera microscope was used in our study to capture the images.

The macroscopic and microscopic analysis of the resection specimens revealed chronic exo-cervicitis with signs of viral infection, chronic micropapillary endo-cervicitis, hyperplasic endometrial polyp, adenomyosis, follicular ovarian cysts, multiple para tubal cysts, and an immunohistochemically confirmed cervical epidermoid cyst.

The endo-cervix was lined by a single layer of columnar mucous cells and had an micropapillary architecture, moderate inflammatory infiltrate in the surface chorion, and areas of squamous metaplasia and moderate inflammatory infiltrate in the surface chorion in some other parts could be seen.

**Figure 1:**
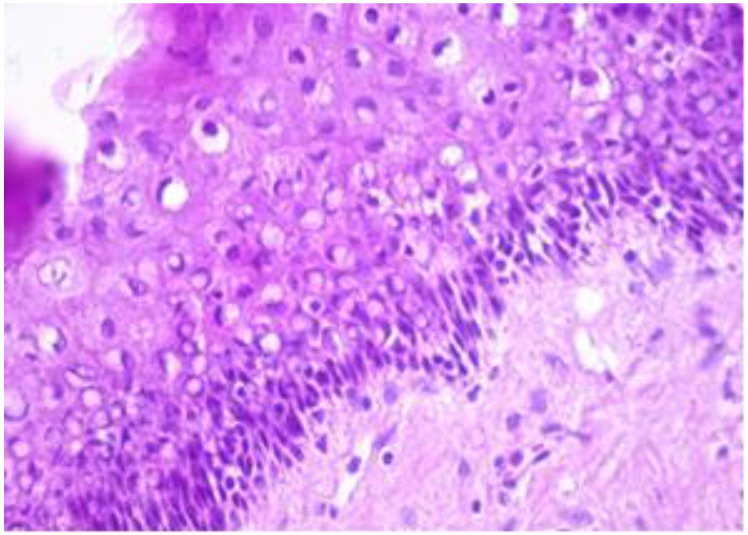
Exo-cervix showing a distinct basal layer and atypical keratinocytes (HE staining, 40×).

**Figure 2:**
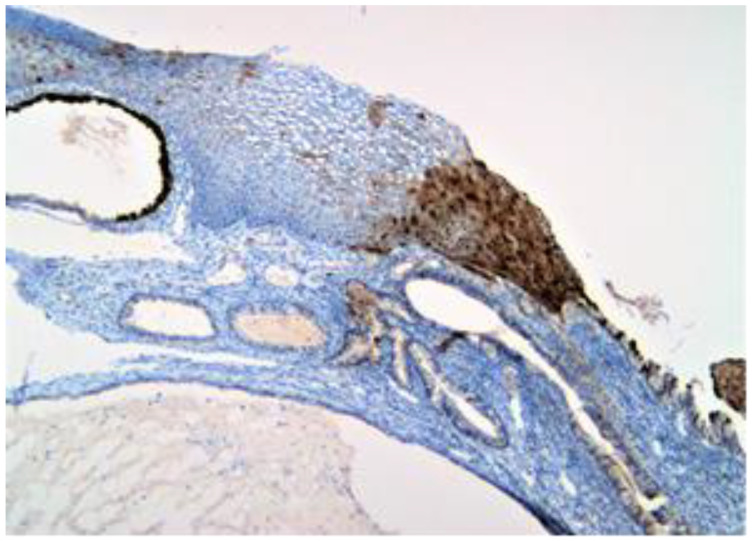
p16 positive (brown) expression to the nuclear and cytoplasmic level in the entire thickness of the mucosa.

The exo-cervix was found to be lined by a non-keratinised stratified epithelium (squamous epithelium) showing a distinct basal layer, but the appearance of atypical keratinocytes was noted, with some of them showing koilocytotic atypia with sharply outlined perinuclear vacuoles, dense and irregular staining peripheral cytoplasm and an enlarged, modified nucleus (Figure 1). The p16 expression showed a positive nuclear/cytoplasmic expression in the cervical mucosa (Figure 2). 

**Figure 3:**
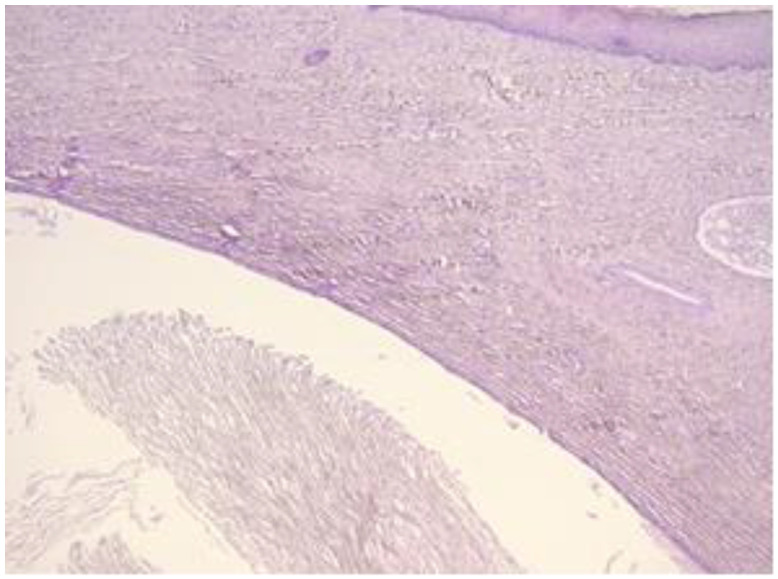
Exo-cervix shows a cystic structure in the deeper layers that is filled with an amorphic, eosinophilic, lamellar substance (HE staining, 5×).

**Figure 4:**
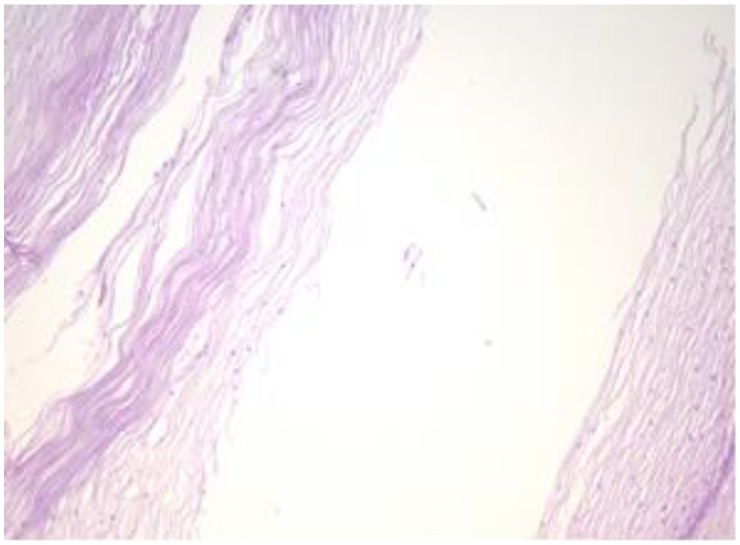
Cyst is lined by a stratified epithelium that resembles a squamous epithelium, similar in appearance to the endo-exocervical junctional area (HE staining, 10×).

The exo-cervix shows a cystic structure in the deeper layers that is filled with an amorphic, eosinophilic, lamellar substance (Figure 3). The cyst is lined by stratified epithelium resembling squamous epithelium that reminds us of the typical structure found at the level of the endo-exocervical junction (Figure 4).

**Figure 5:**
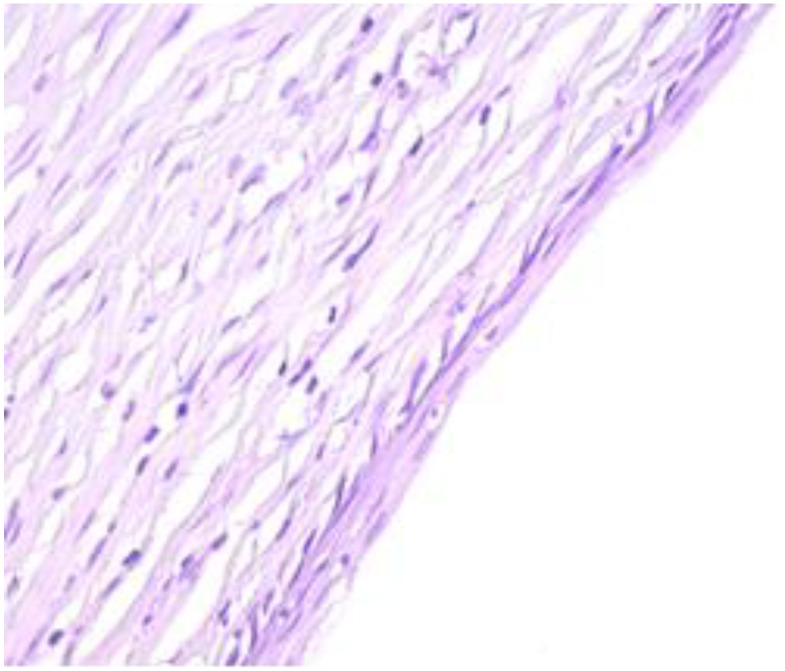
Other areas of the cyst are lined by a stratified, flattened epithelium with focally outer fine, but distinct, granulated eosinophilic cytoplasm cells (HE staining, 40×).

**Figure 6:**
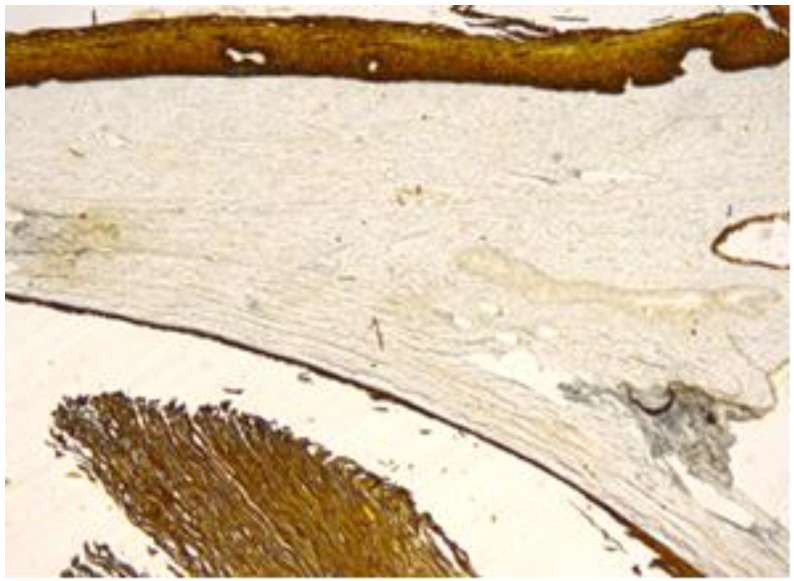
Cyst’s epithelium, content and overlying exocervical epithelium, marked positive (CK 5/6, 10×).

**Figure 7:**
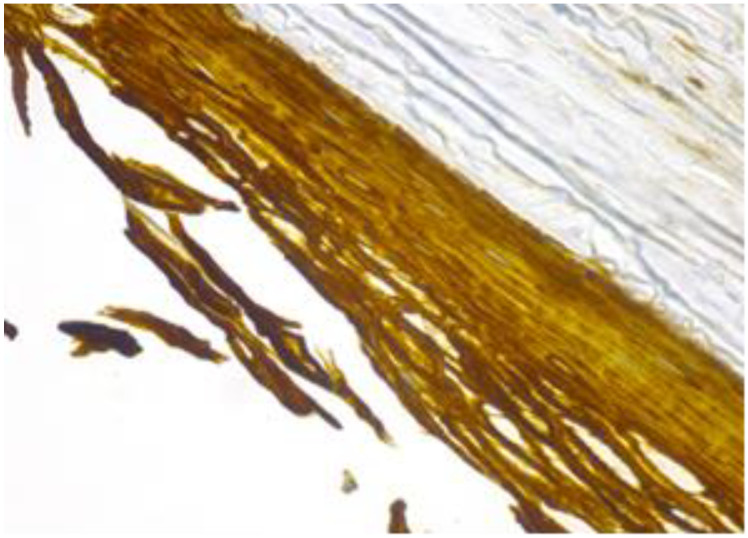
Cyst’s epithelium marked as intensively positive (CK 5/6, 40×).

Other areas of the cyst are lined by stratified, flattened epithelium with focally outer fine, but distinct, granulated eosinophilic cytoplasm cells (Figure 5). The cyst’s epithelium, content and overlying exocervical epithelium are marked positive (Figure 6). The epithelium of the cyst is also marked as intensely positive (Figure 7). The myometrium presents islands of endometrial stromal and glandular structures within its thickness. In the endometrium, we are able to assess the ratio between the glandular structures and stroma and we notice that it is strongly modified in the gland’s favour. Para tubal, we see a cystic structure with amorphic, slightly eosinophilic content. This cystic formation is lined at the most intimate level by a single layer of cuboidal, cylindrical cells, sometimes presenting a flattened structure.

## 3. Discussion

The cervical localisation of an epidermal-type keratinocyte cyst is particularly unusual. The immunohistochemical studies of the specimens proved without any doubt that the cystic structure was indeed an epidermal cyst and the etiopathology of an epidermoid cyst with “classical” localisation is probably very well understood. In this case, the etiopathology of such a lesion located in the cervix is an ongoing debate. Some causes of it could be: local irritating conditions such as trauma, chronic inflammation, infection with HPV, squamous metaplasia, growth originating from vestigial structures and heterotrophic intrapartum implants. The exact etiopathology is unknown, although animal research studies suggested that reminiscent vestigial structures are likely candidates to induce such a lesion [17]. The uterus, cervix and upper two-thirds of the vagina develop embryologically from the paired Müllerian ducts from their fused distal portion [23,24].

Despite the fact that epidermoid cysts are commonly seen localised in hair-bearing skin, diagnosing them at mucosa sites, such as in our case, is extremely rare. The association between HPV infection and the epidermoid cyst presence has been debated in many studies [25,26]. High-risk HPV strains are proven to be a carcinogenetic factor in the case of cervical cancer. Other studies try to determine whether there is any possible connection between HPV infections and malignant transformations in cases of mature cystic teratomas. Chiang et al. [27] demonstrated that HPV might induce the transformation of ovary teratoma in squamous cell carcinoma. Many studies performed in the last decade demonstrated the role played by high-risk HPV strains (16, 18, 31, 33, 34, 35, 39, 45, 51, 52, 56, 58, 59, 66, 68 and 70) in the cervix cancer pathogenesis. One of the more exhaustive studies regarding the HPV role stated that 6, 11, 16, 30, 33, 36, 37, 38, 41, 48, 60, 72 and 73 subtypes might be found in the skin layer of epidermoid cysts [26,28,29,30]. A Korean study [9] showed that there is in fact an association between HPV 57 and 60 and the occurrence of palmoplantar epidermoid cysts. 

In our study, we also tried to assess whether there was any HPV expression in the samples of the cervix and/or epidermoid cyst that we had diagnosed. The positive p16 nuclear and cytoplasmic expression was detected, in our case, at the level of the exo-cervix, but it was revealed to be totally negative in the epidermoid cyst. The negative result only excluded high-risk HPV strains and thus left enough space for other HPV subtypes to be evaluated.

Moreover, p16 plays an important role in the cell cycle as a cyclin-dependent kinase inhibitor; p16 acts like an indirect marker for the HPV infection and the overexpression is induced by viral E7 protein [31]. The malignant transformation of the epidermoid cyst in squamous cell carcinoma or other skin cancer types is rarely encountered [32,33,34,35,36,37,38,39]. 

According to Stolnicu et al. [40], very few cases of teratomas located in the cervix and uterus are reported in the medical literature. In most of the cases, the authors considered the phenomenon to occur because of foetal tissue implantation or abortions [41,42]. Thus, we can assume that, as an origin, these teratomas derive (including the epidermoid cyst described here) from pluripotential stem cells [40].

In our case, we also tried to find other cysts whose presence could be explained on the basis of a genetically determined background, but in the end, there was actually no evidence of multiple epidermoid cysts, so this cyst most probably had a sporadic nature.

## 4. Conclusions

The exact etiopathology of the epidermoid cyst is unknown, but reminiscent vestigial structures are a likely candidate to induce such lesions. Further studies should be performed in order to assess the recurrence rate or even malignancy transformation potential, since an increasing number of cases are being reported at unusual sites such as the genital areas. The association between any HPV subtypes and epidermoid cysts is still a debated issue because its pathology is extremely rarely encountered.

## Data Availability

Data available on request.
